# Evaluating the association between opium abuse, blood lead levels, and the complexity of coronary artery disease

**DOI:** 10.14814/phy2.15975

**Published:** 2024-03-13

**Authors:** Ramin Khameneh Bagheri, Seyed Hadi Mousavi, Hassan Mehrad‐Majd, Mohammad Javad Jamili, Arya Nasimi Shad, Vafa Baradaran Rahimi

**Affiliations:** ^1^ Department of Cardiovascular Diseases, Faculty of Medicine Mashhad University of Medical Sciences Mashhad Iran; ^2^ Medical Toxicology Research Center Mashhad University of Medical Sciences Mashhad Iran; ^3^ Clinical Research Development Unit, Ghaem Hospital Mashhad University of Medical Sciences Mashhad Iran; ^4^ Student Research Committee, Faculty of Medicine Mashhad University of Medical Sciences Mashhad Iran; ^5^ Pharmacological Research Center of Medicinal Plants Mashhad University of Medical Sciences Mashhad Iran

**Keywords:** blood lead level, coronary artery disease, opium, SYNTAX I score

## Abstract

Opium abuse and exposure to heavy metals elevate the risk of coronary artery disease (CAD). Therefore, we aimed to determine the association between opium abuse and blood lead levels (BLLs) and the CAD complexity. We evaluated patients with acute coronary symptoms who underwent coronary angiography, and those with >50% stenosis in at least one of the coronary arteries were included. Furthermore, Synergy between PCI with Taxus and Cardiac Surgery I (SYNTAX I) score and BLLs were measured. Based on the opium abuse, 95 patients were subdivided into opium (45) and control (50) groups. Differences in demographics and CAD risk factors were insignificant between the two groups. The median BLLs were remarkably higher in the opium group than in controls (36 (35.7) and 20.5 μg/dL (11.45), respectively, *p* = 0.003). We also revealed no significant differences in SYNTAX score between the two groups (15.0 (9.0) and 17.5 (14.0), respectively, *p* = 0.28). Additionally, we found no significant correlation between BLLs and the SYNTAX scores (*p* = 0.277 and *r* = −0.113). Opium abuse was associated with high BLLs. Neither opium abuse nor high BLLs were correlated with the complexity of CAD. Further studies are warranted to establish better the relationship between opium abuse, BLLs, and CAD.

## Graphical abstract

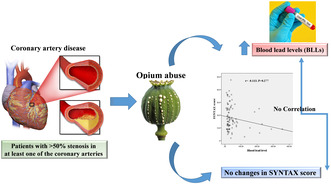

## 1 INTRODUCTION

Cardiovascular diseases (CVDs), especially coronary heart diseases (CHDs), are a major cause of morbidity and deaths globally (Gholoobi et al., 2021). Various traditional risk factors are associated with a higher incidence of CHDs, such as diabetes mellitus, obesity, hypertension, unhealthy diet, low physical activity, smoking, and dyslipidemia (Khameneh Bagheri et al., 2022; Wong, 2014). Due to the persistent prevalence of CVDs, ongoing research is being conducted on possible novel CVD risk factors, including opium abuse and environmental hazards such as heavy metals (Lanphear et al., 2018; Masoudkabir et al., 2013).

World Drug Report estimated that over 53.4 million individuals misused different opioids and opiates in 2017, making opioids and opiates among the most commonly abused illicit drugs worldwide (Drugs, U.N.O. and Crime, 2019). The issue is also even more substantial in Asia and the Middle East since the two largest opium‐producer countries (Afghanistan and Myanmar) are located in this region (Drugs, U.N.O. and Crime, 2019). Opium is principally isolated from *Papaver somniferum* capsule seeds. It has been reported that different source of poppy seeds possess different opium alkaloids, such as morphine, papaverine, codeine, thebaine, and noscapine which may be responsible for their different effects (Carlin et al., 2020).

There are inconsistencies regarding the impacts of opioid abuse on coronary artery disease (CAD) and its potential detrimental or beneficial effects. There are numerous beneficial aspects of opioid therapy during chronic devastating pains such as cancer (Bruera & Paice, 2015), chronic kidney disease (Tobin et al., 2022), and osteoarthritis (Altman & Smith, 2010). Multiple studies reported opium abuse as an independent risk factor for CAD (Bahrami et al., 2021; Maino et al., 2023), while others found no significant relationship between these two entities (Masoumi et al., 2010; Rezvani & Ghandehari, 2012).

It has been demonstrated that multiple heavy metals such as cadmium, mercury, and lead may potentially deteriorate cardiovascular health (Choi et al., 2020). It is suggested that blood lead level (BLL) is an independent risk factor for CVDs. Additionally, a significant correlation was seen between BLL and several traditional cardiovascular (CV) risk factors, such as fasting plasma glucose (FPG), body mass index (BMI), and high blood pressure (Chen et al., 2017). However, it is still unclear whether there is a causal relationship between lead exposure and CAD or whether it occurs due to other potential confounding factors such as poor lifestyle and low socioeconomic level (Schooling et al., 2019).

Exposure to heavy metals such as lead may occur through various routes, such as water pollution, foods and plants grown on polluted soil, occupational exposure, and lead impurities found in opioids (Asgary et al., 2017; Etemadi et al., 2022). Asgary and colleagues have suggested the potential presence of a correlation between CHDs and long‐term exposure to even minor amounts of lead (Asgary et al., 2017). A high BLL is seen among Iranian opium addicts (Etemadi et al., 2022), which is also coherent with the presence of remarkable lead contamination in opium products (Aghababaei et al., 2018).

As we mentioned earlier, CAD, opium abuse, and exposure to heavy metals all contribute to major public health issues globally. Therefore, this study is designed to evaluate the correlation between opium abuse and BLLs, as well as the impacts of opium abuse and BLLs on the complexity of CAD.

## 2 METHODS

### 2.1 Ethical consideration

The ethics committee of Mashhad University of Medical Sciences approved this study with the approval code of IR.MUMS.MEDICAL.REC.1400.583. In addition, written informed consent was obtained from all subjects.

### 2.2 Study design and participant selection

The present study was conducted in Ghaem Hospital, affiliated with the Mashhad University of Medical Sciences, from November 2021 to May 2023. The study population comprised patients who were admitted to the hospital, either electively or urgently, with signs and symptoms of acute coronary syndrome (ACS) and underwent coronary angiography. The inclusion criteria were to have >50% stenosis in at least one of the coronary arteries found in coronary angiography. An expert interventional cardiologist carried out all coronary angiography procedures.

Exclusion criteria include (1) older than 75 years, (2) positive family history of CVDs, (3) patients with chronic kidney disease, (4) patients who withdrew opium abuse or were addicted to opium derivatives or other recreational drugs, (5) patients who have occupations with lead exposure, and (6) absence of consent.

### 2.3 Outcome evaluation

All individuals filled out a written questionnaire that covered the patient's medical history and demographics such as age, gender, place of residence (rural or urban), family history, working environment, history of opium abuse, route of opium abuse, cigarette smoking, and history of diabetes mellitus (DM), hypertension (HTN), or dyslipidemia (DLP). In addition, the history of hypertensive, diabetic, or dyslipidemia was addressed according to the physicians diagnosis recorded in the hospital medical records. In fact, we recorded the history of diabetes mellitus, hypertension, and dyslipidemia which was documented in the hospital medical records by an expert physician according to the patient's medical history. As an example, the history of diabetes was diagnosed by a physician according to taking anti‐diabetes medications at the time of referral to the hospital.

Patients were then subdivided into two groups according to their history of opium abuse. In addition, the SYNTAX I scores, and blood lead levels were determined and documented for further analysis.

### 2.4 SYNTAX I score

The complexity of CAD was evaluated by the Synergy between PCI with Taxus and Cardiac Surgery I (SYNTAX I) score, which is a proven angiographic tool to estimate the extent of CAD complexity and guide clinicians to decide between the percutaneous coronary intervention (PCI) and coronary artery bypass graft (CABG) (Farooq et al., 2014). An expert interventional cardiologist conducted angiographic procedures, and the angiograms and SYNTX score results were documented. SYNTAX I scores were calculated using www.syntaxscore.org (Safarian et al., 2014).

### 2.5 Sample collection and blood lead analysis (BLL)

Blood samples (2 mL) were collected from each individual during hospital admission to determine BLLs, which were measured using Varian SpectrAA 220 atomic absorption spectrophotometer.

### 2.6 Statistical analysis

Data were analyzed using the SPSS version.25 statistical software (SPSS Inc., Chicago, Illinois) and GraphPad Prism 8.01 software (GraphPad Software Inc., USA). Moreover, data were shown according to parametric and non‐parametric nature as means ± SD, median with interquartile range (IQR), or number with percentage, respectively. The variables were compared using an independent *t*‐test for parametric data or Mann–Whitney *U*‐tests for non‐parametric data. The comparison between categorical variables was performed using the Chi‐square test. In addition, the correlation between BLL and SYNTAX scores was measured using the Pearson and Spearman correlation coefficients for normally and non‐normally distributed data, respectively. The *p‐*values ≤ 0.05 levels were considered statistically significant.

## 3 RESULTS

### 3.1 Demographics and coronary artery disease risk factors

A total number of 95 patients were recruited based on the aforementioned inclusion and exclusion criteria. Patients were subdivided into two groups according to their history of opium abuse, in which 45 patients were considered opium group and 50 individuals were subjected as controls. Among opium group, 13 (28%) patients abuse orally, 23 (51.0%) smoked, and 9 (20.0%) patients used both orally and smoked routes.

Table 1 demonstrates the demographics and risk factors variability in the opium group and controls. Accordingly, the characteristics of patients in both the opium and control groups were insignificant regarding determined variables such as age, gender, place of residence, history of smoking, DM, HTN, and DLP.

**TABLE 1:** Demographic characteristics of enrolled patients.

Variables	Opium (*N* = 45)	Control (*N* = 50)	*p*‐value
Age (mean ± SD)	57.36 ± 10.14	60.76 ± 7.83	0.069^a^
Gender (*N*, %)			
Male	36 (80%)	32 (64%)	0.084^b^
Female	9 (20%)	18 (36%)	
Place of residence (*N*, %)			
Rural	20 (44.4%)	26 (52%)	0.462^b^
Urban	25 (55.6%)	24 (48%)	
Smoking (*N*, %)	13 (28.9)	8 (16%)	0.131^b^
DM (*N*, %)	17 (37.8%)	18 (36%)	0.858^b^
HTN (*N*, %)	19 (42.2%)	28 (56%)	0.180^b^
DLP (*N*, %)	13 (28.9)	14 (28%)	0.924^b^

Abbreviations: DLP, dyslipidemia; DM, diabetes mellitus; HTN, hypertension.

^a^ Comparing the results of opium and control groups using independent samples *t*‐test.

^b^ Comparing the results of opium and control groups using Chi‐square test.

The mean age of opium and controls were 57.36 ± 10.14 and 60.76 ± 7.83, respectively (*p* = 0.069). Females comprised 28.4%, whereas males constituted 71.6% of enrolled individuals. A total of 49 participants (51.6%) lived in urban areas, while 46 patients (48.4%) lived in rural regions, and differences between these two groups were insignificant (*p* > 0.05).

### 3.2 BLL and opium abuse

**FIGURE 1:**
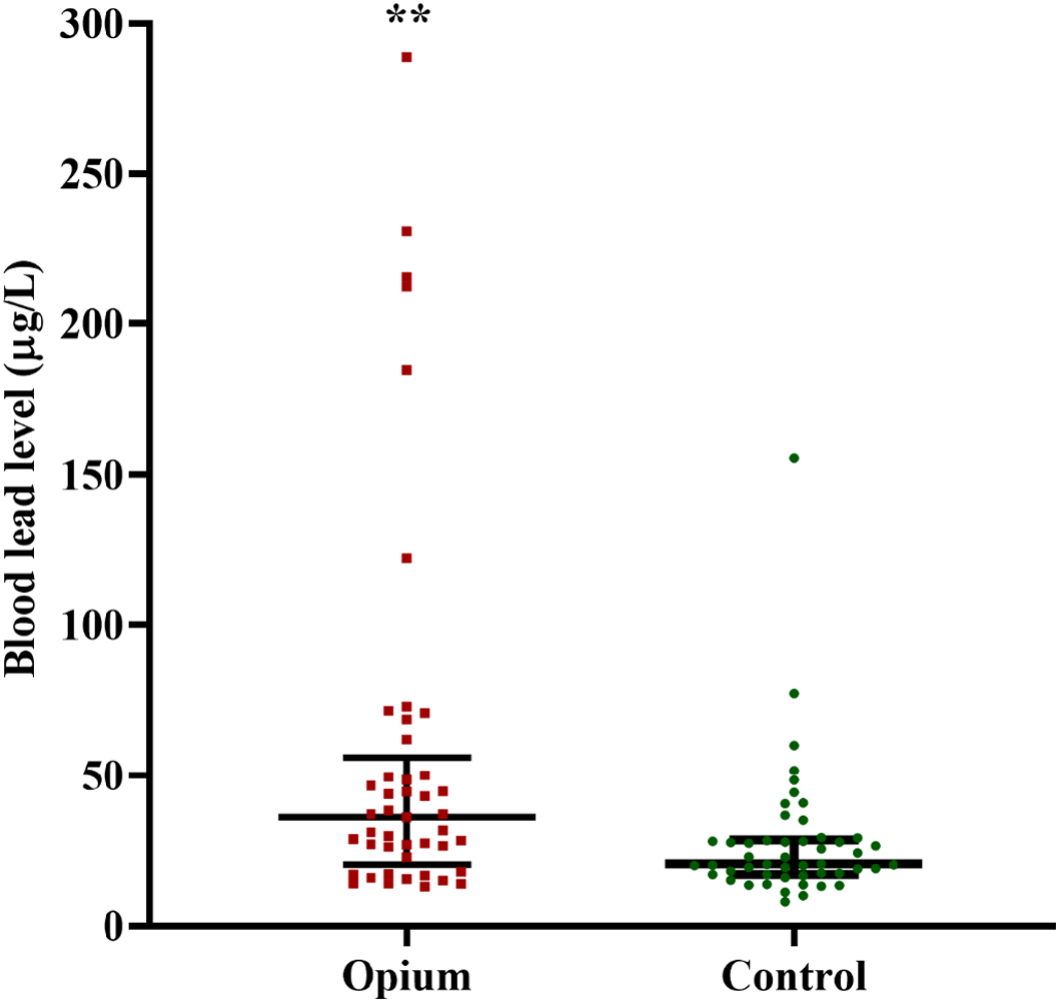
The results of BLL in patients with coronary artery disease with (opium group) and without (control) opioid use; Data were presented as median and interquartile range (IQR); Comparison was done using Mann–Whitney *U*‐test; **p = 0.003 between the two studied groups.

The BLLs of opium and control groups are illustrated in Figure 1. The opium group showed a median BLL of 36 μg/dL (IQR = 35.7), whereas the median BLL in the control group was 20.5 μg/dL (IQR = 11.45). Based on the Mann–Whitney test, a significantly higher BLL was seen in the opium group than in the control group (*p* = 0.003, Figure 1).

### 3.3 Opium abuse and SYNTAX score

**FIGURE 2:**
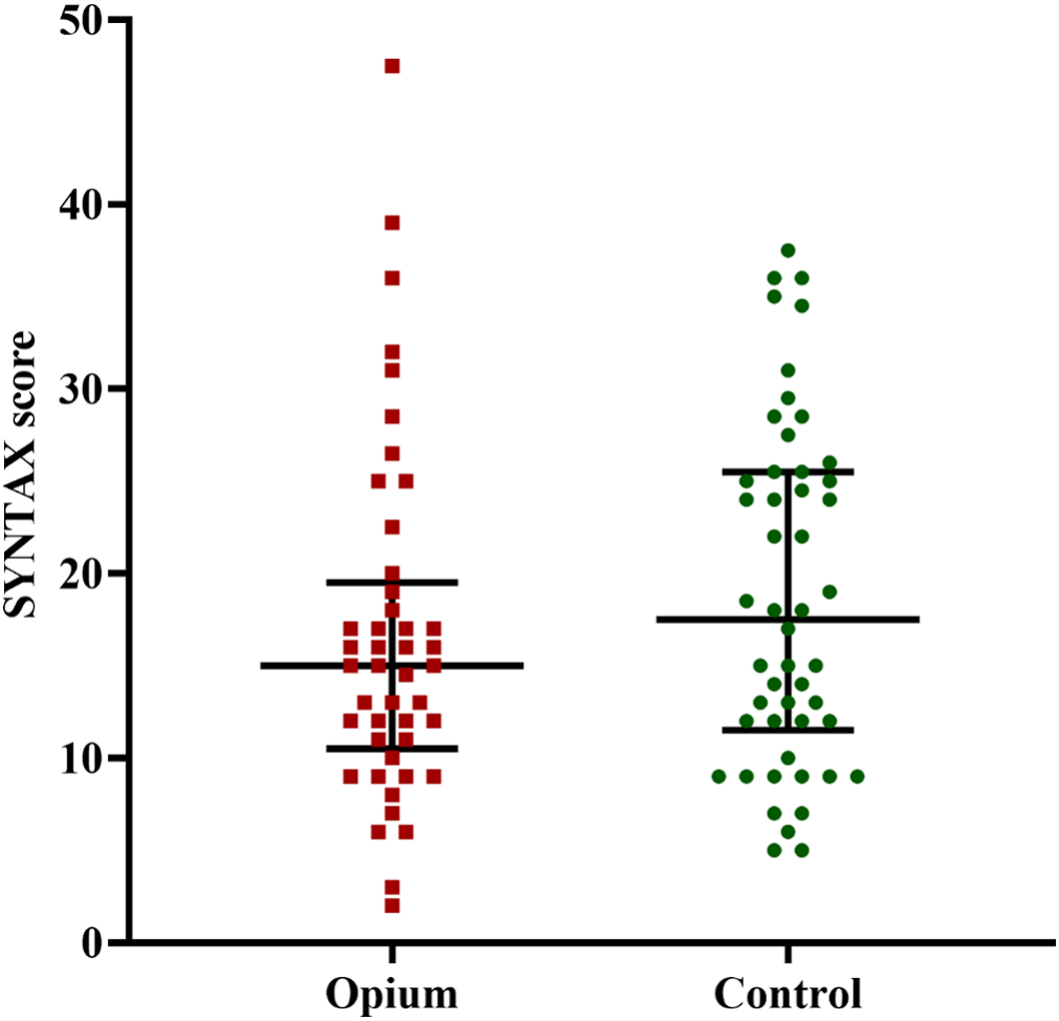
The results of SYNTAX score in patients with coronary artery disease with (opium group) and without (control) opioid use; Data were presented as median and interquartile range (IQR); Comparison was done using Mann–Whitney *U*‐test. *p* = 0.28 between the two studied groups.

Figure 2 illustrates the non‐normal distribution of SYNTAX scores in the opium group and controls. Based on the documented angiograms, the SYNTAX score was 15.0 (IQR = 9.0) and 17.5 (IQR = 14.0) in the opium and control group, respectively (*p* = 0.28). Although the opium group had a lower median SYNTAX score than the controls, the difference was insignificant (*p* = 0.28, Figure 2).

### 3.4 The correlation between BLL and SYNTAX score

**FIGURE 3:**
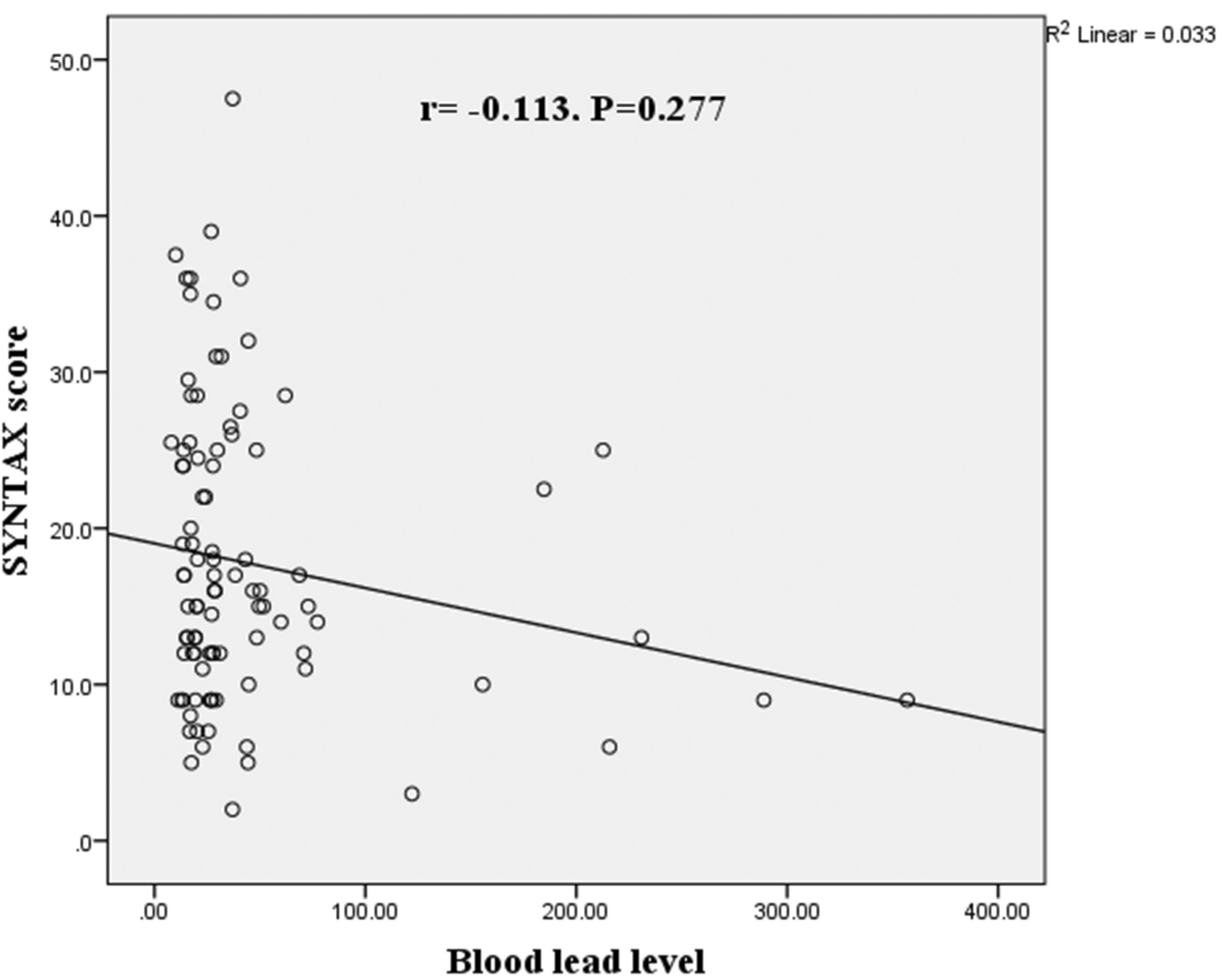
Correlation between BLL and SYNTAX score in studied patients; Correlation was measured using the Spearman correlation coefficients. Correlation coefficient = − 0.113 and *p* = 0.277.

According to Spearman's correlation coefficient, there was no significant correlation between BLLs and the SYNTAX scores, which revealed a *p*‐value of 0.277 and *r* = − 0.113. Figure 3. Demonstrates the correlation between BLLs and SYNTAX scores.

## 4 DISCUSSION

In the present study, we measured the complexity of coronary artery disease, history of opium abuse, and BLL in CAD patients. The association among these variables demonstrated that opium abuse significantly accompanies higher BLLs. However, we did not find a significant association between opium abuse and the complexity of CAD, which was consistent with previous studies (Marmor et al., 2004; Rezvani & Ghandehari, 2012). However, several studies reported incompatible results, illustrating that opium addiction was significantly associated with a higher risk of CAD (Maino et al., 2023; Masoomi et al., 2010). We also established that higher BLL is not correlated with the higher SYNTAX scores and CAD complexity.

Our results revealed that opium abuse is significantly accompanied by higher BLLs. Significantly high lead contamination was seen in various illicit drugs such as opium, heroin, and crack (Aghababaei et al., 2018). The exact route of lead contamination is still unclear, but it might be due to polluted oil or water used during farming opium products. It is important to note that drug traffickers may also add heavy metals such as lead to opium products for financial purposes (Aghababaei et al., 2018). In a recent cohort study conducted in northeast Iran, Etemadi et al. showed an independent dose‐response correlation between BLLs and the weekly dose of long‐term oral or smoked opium abuse (Etemadi et al., 2022). Hayatbakhsh and colleagues also found meaningfully higher BLLs in opium‐addicted individuals, which was surprisingly independent of the duration of use, daily dosing, route of use, and type of substance (Hayatbakhsh et al., 2017). These results are in line with our results regarding the higher BLLs in opium‐addicted patients.

In the present study, we enrolled patients with >50% stenosis in at least one of the coronary arteries among ACS patients who underwent coronary angiography. In addition, we excluded a positive family history of CVDs. We categorized patients according to opium abuse or not. We revealed that although the opium group had a lower median SYNTAX score than the controls, the difference was insignificant. There is an enduring debate on the association of opiates with the risk and prognosis of CAD. Marmor et al. have shown that opiate consumption is negatively associated with CAD severity and complications. They supported that patients with opium abuse had less often severe CAD than the non‐opium group (OR = 0.32, 95% CI = 0.1–0.9) (Marmor et al., 2004). Rezvani et al. found neither harmful nor preventive roles for oral or inhaled opium abuse in ischemic heart disease. However, they suggested a protective role for oral opium against ischemic stroke (OR = 0.211, 95% CI = 0.079–0.56) (Rezvani & Ghandehari, 2012). Ogungbe et al. conducted a cross‐sectional study that included 1829 patients and found no significant relationship between prescribed opioid use and CAD (Ogungbe et al., 2019). Similarly, long‐term prescription opioid use was not accompanied by coronary heart disease or stroke (Khodneva et al., 2016).

In contrast to the protective or not‐harmful effects of opium on CAD, some studies reported the stimulated risk of CAD in opium consumers. Recently, Maino and coworkers noticed habitual opium consumers have a significantly elevated risk of experiencing CAD. They also reported that hyperlipidemia may have a supra‐additive correlation with opium consumption regarding CAD (Maino et al., 2023). Masoomi et al. also reported that opium abuse is an independent risk factor for CAD. They reported a higher number of opium abuse in CAD patients than in subjects with normal coronary arteries (Masoomi et al., 2010). These studies compared CAD patients with normal coronary artery subjects and did not exclude patients with positive family history of CVDs.

Opioids are frequently used as potent analgesics during acute or chronic pains. Opium is composed of alkaloid and non‐alkaloid components. Alkaloid compounds mostly include Morphine, Codeine, Papaverine, Thebaine, Narceine, and Noscapine, whereas non‐alkaloid components include sugar, water, and various simple organic acids (Khademi et al., 2016).

The main mechanisms that opium consumption affects CAD are yet to be determined. Several pieces of evidence suggest that opioid medications may have cardio‐protective effects. Indeed, activation of opioid receptors, including kappa‐opioid receptors (KOR), delta opioid receptors (DOR), and mu opioid receptors (MOR) inhibited myocardial ischemia‐reperfusion injury (IRI) and cardiomyocyte death (Roth et al., 2021). Noteworthy, MOR is generally absent in healthy and non‐ischemic hearts; however, it specifically upregulated in ischemic and heart failure conditions (Jin et al., 2018). It has been suggested that non‐coding RNAs are responsible for the inhibition of apoptosis and protective effects of opioids against IRI (Melo et al., 2018). In fact, the opioid receptor system protects against all major IR mechanisms, including apoptosis, contractile dysfunction, arrhythmogenesis, and inflammation (Baradaran Rahimi & Askari, 2022; Headrick et al., 2015). Therefore, the insignificant lower SYNTAX score in the opium group than the controls may be due to the cardio‐protective effects of opium.

Our results also showed that higher BLLs were not correlated with the higher SYNTAX scores and CAD complexity. Li and coworkers evaluated 343 patients with CAD and found that BLLs were correlated with intermediate and high SYNTAX scores. They examined emergency ACS patients and, not including elective patients with stable angina symptoms. This issue causes the SYNTAX score of these patients to be calculated higher due to the higher chance of thrombosis and complete obstruction. They also did not exclude the positive familial cardiovascular diseases that can notably affect the SYNTAX score (Li et al., 2021). Kim et al. investigated 2193 adults without cardiovascular disease or occupational exposure to lead. They suggested that BLLs were remarkably accompanied by moderate‐to‐severe coronary artery stenosis. However, they used CT‐angiography to examine the complexity of coronary artery stenosis rather than angiography, which is the golden standard. In addition, they did not exclude the family history of cardiovascular disorders (Kim et al., 2021).

Multiple studies have shown a significant correlation between unfavorable lead exposure and elevated CVDs (Asgary et al., 2017; Chen et al., 2017). Indeed, the negative effects of high BLLs are undeniable. It has been suggested that long‐term exposure to even minor amounts of lead could potentially negatively affect CV health (Asgary et al., 2017). However, none of these studies evaluated BLLs in opium consumers. Therefore, the insignificant relation between BLLs and SYNTAX score in our study may be due to the cardioprotective effects of opium. In addition, the exact toxic dose of BLLs in terms of CVDs, especially CAD, is still not determined, which may be the second reason for our insignificant results. Although we did not find a significant correlation, given the aforementioned data on how opium and BLL may affect the incidence or prognosis of CAD, there is a major need for high‐quality, multi‐center, and prospective data on this issue.

### 4.1 Limitations

Our study holds several limitations. First, we used a self‐reported method to evaluate opium abuse. However, it was shown that self‐reported data regarding opium abuse is a reliable method in the Iranian population (Abnet et al., 2004). It is recommended to select opium addict and non‐opium addict patients according to blood test in order to confirm the opium addiction. Second, we performed a single‐center study with a small sample size. It is recommended to perform multi‐center studies with a larger population to understand better the exact effects and mechanisms of opium and BLLs on the complexity of CAD. Third, as there was a lack of proper data, we could not calculate the dose‐response interrelation between opium abuse, BLL, and CAD complexity. Moreover, our cross‐sectional study makes it difficult to draw a causal relationship between the analyzed variables. Finally, we didn't documented and reported the BMI, levels of systolic and diastolic blood pressure, plasma lipids, and plasma sugar which are very critical in patients with coronary artery disease. To better understand the discussed issue, we recommend future research studies to prospectively evaluate the dose‐response correlation between opium abuse and BLLs with the risk and prognosis of CAD.

## 5 CONCLUSION

Opium abuse was correlated with higher BLLs; however, there was no relationship between opium abuse and the complexity of CAD. In addition, we revealed no association between BLLs and CAD complexity. Further prospective studies with larger sample sizes are required to establish better the impact of opium and various BLLs on CAD.

## AUTHOR CONTRIBUTIONS

Ramin Khameneh Bagheri: conceptualisation, methodology, supervision, investigation; Seyed Hadi Mousavi: conceptualisation, supervision, funding acquisition; Hassan Mehrad‐Majd: formal analysis, data curation; Mohammad Javad Jamili: investigation, data curation; Arya Nasimi Shad: investigation, writing—original draft, writing—review & editing; Vafa Baradaran Rahimi: formal analysis, writing—original draft, writing—review & editing.

## FUNDING INFORMATION

This study was financially supported by grant number 980721 from Mashhad University of Medical Sciences.

## CONFLICT OF INTEREST STATEMENT

The authors declare no conflict of interest.

## ETHICS STATEMENT

The ethics committee of Mashhad University of Medical Sciences approved this study with the approval code of IR.MUMS.MEDICAL.REC.1400.583. In addition, written informed consent was obtained from all subjects.

## Data Availability

The data sets used for the current study are available from the corresponding author upon reasonable request.
